# Network Pharmacology and Molecular Docking Analyses Unveil the Mechanisms of Yiguanjian Decoction against Parkinson's Disease from Inner/Outer Brain Perspective

**DOI:** 10.1155/2022/4758189

**Published:** 2022-09-26

**Authors:** Zhongqi Shen, Meng Yu, Shaozhi Zhang

**Affiliations:** ^1^College of Traditional Chinese Medicine, Shandong University of Traditional Chinese Medicine, Jinan, Shandong, China 250355; ^2^Innovative Institute of Chinese Medicine and Pharmacy, Shandong University of Traditional Chinese Medicine, Jinan, Shandong, China 250355

## Abstract

**Objective:**

This study aims to explore the pharmacodynamic mechanism of Yiguanjian (YGJ) decoction against Parkinson's disease (PD) through integrating the central nervous (inner brain) and peripheral system (outer brain) relationship spectrum.

**Methods:**

The active components of YGJ were achieved from the TCMSP, TCMID, and TCM@Taiwan databases. The blood-brain barrier (BBB) permeability of the active components along with their corresponding targets was evaluated utilizing the existing website, namely, SwissADME and SwissTargetPrediction. The targets of PD were determined through database retrieval. The interaction network was constructed upon the STRING database, followed by the visualization using Cytoscape software. Then, we performed Gene Ontology (GO) and Kyoto Encyclopedia of Genes and Genomes (KEGG) enrichment analyses on potential targets. Finally, the molecular docking approach was employed to assess the binding affinity between key components and key targets.

**Results:**

Overall, we identified 79 active components, 128 potential targets of YGJ, and 97 potential targets of YGJ-BBB potentially suitable for the treatment of PD. GO and KEGG analyses showed that the YGJ treatment of PD mainly relied on PI3K-Akt pathway while the YGJ-BBB was mostly involved in endocrine resistance. The molecular docking results displayed high affinity between multiple compounds and targets in accordance with previous observations.

**Conclusions:**

Our study unveiled the potential mechanisms of YGJ against PD from a systemic perspective: (1) for the YGJ, they have potential exerting effects on the peripheral system and inhibiting neuronal apoptosis through regulating the PI3K-Akt pathway; (2) for the YGJ-BBB, they can directly modulate endocrine resistance of the central nervous and holistically enhance body resistance to PD along with YGJ on PI3K-Akt pathway.

## 1. Introduction

Parkinson's disease (PD) is the second most common neurodegenerative disease in the world [1]. Its pathology is characterized by the loss of neurons in the substantia nigra leading to a decrease in dopamine (DA) transmitters in the striatum and the formation of Lewy bodies containing *α*-synuclein (*α*-syn) [2]. Clinically, symptoms of PD include typical motor symptoms (bradykinesia, rigidity, resting tremor, and gait disturbances) and non-motor symptoms (sleep disturbances, olfactory disturbances, autonomic dysfunction, and cognitive and psychiatric disturbances) [3]. Currently, levodopa is the standard of care for PD treatment. However, levodopa does not completely cure PD, and long-term treatment is often accompanied by side effects [4]. Therefore, the research of alternative drugs is of great importance for PD treatment.

In Traditional Chinese Medicine (TCM) theory, patients with PD often have the symptom of yin deficiency of the liver and kidney [5, 6]. Classically, the Yiguanjian (YGJ) decoction is the standard prescription to treat disease of this syndrome type [7, 8], including Beishashen (*Glehniae Radix*, root), Chuanlianzi (*Toosendan Fructus*, fruit), Danggui (*Angelicae Sinensis Radix*, root), Dihuang (*Rehmanniae Radix Praeparata*, root), Gouqizi (*Lycii Fructus*, fruit), and Maidong (*Ophiopogon japonicus*, root) [9, 10].

Although the YGJ has obvious therapeutic effect against PD, there is still no systematic study about this. Network pharmacology, as part of bioinformatics technology, integrates systems biology and computational biology [11]. With the rise of technologies such as molecular docking, molecular dynamics simulations, and bioinformatics, in silico strategy has emerged. It can reveal the relationship between molecular monomers with biological pathways or specific diseases by comprehensively studying and expanding the intersection between molecular monomers [12–14]. Network pharmacology allows the relationships between TCM and disease to be explored as a whole, and the mechanisms between TCM and disease can be systematically revealed and thus provide a systematic approach for the study of TCM treatment of diseases [15]. According to this, we conducted this study to explore the therapeutic mechanisms of YGJ against PD for the first time through the approach of network pharmacology and molecular docking.

**Figure 1:**
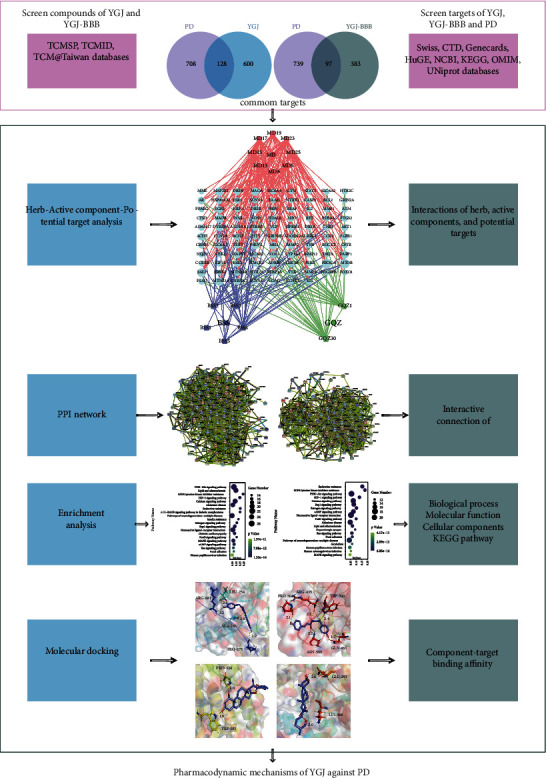
Diagrammatic illustration of the workflow of the study.

In our approach, we assigned all active components to the YGJ group and placed the active components crossing the BBB to the YGJ-BBB group. Through this operation, we can not only explore the effects of all active components of YGJ on peripheral system (outer brain) but also screened out the components that can cross the BBB to specifically explore the therapeutic effects of YGJ on central nervous (inner brain), in order to comprehensively analyze the pharmacodynamic mechanisms of YGJ against PD. Furthermore, a visible graphical abstract for the current flowchart is provided to demonstrate the mechanisms of YGJ against PD concerning central nervous and peripheral systems (Figure 1).

## 2. Methods

### 2.1. Screening of Active Components

The components of YGJ were collected from multiple databases, including the Traditional Chinese Medicine System Pharmacology Database and Analysis Platform (TCMSP, https://old.tcmsp-e.com/tcmsp.php), Traditional Chinese Medicine Integrated Database (TCMID, http://www.megabionet.org/tcmid/), and Traditional Chinese Medicine Database @ Taiwan (TCM@Taiwan, http://tcm.cmu.edu.tw/index.php). Oral bioavailability (OB) is one of the most important parameters of pharmacokinetics, and the higher the OB value, the better the drug-likeness (DL) of the active component [16]. The Caco-2 screening assay is a valuable tool for testing compounds for intestinal permeability [17]. In this study, the criteria of OB ≥30%, DL ≥0.18, and Caco-2 ≥ -0.4 were used to screen the active components from TCMSP [18]. The canonical SMILES of components from TCMID and TCM@Taiwan databases were imported into SwissADME website (http://www.swissadme.ch/) to analyze the bioavailability score. The component with a bioavailability score greater than 0.5 was considered the active component [19, 20]. All active components obtained from the above 3 databases were assigned to the YGJ group.

### 2.2. Screening of Active Components Crossing the BBB

The canonical SMILES of all active components obtained from the above 3 databases were imported into Swiss ADME website to analyze whether they can cross the BBB. The active components crossing the BBB were placed to the YGJ-BBB group.

### 2.3. Acquisition of Targets of PD

Targets of PD were collected from seven databases, including CTD (http://ctdbase.org/), Genecards (https://www.genecards.org/), HuGE (https://phgkb.cdc.gov/PHGKB/hNHome.action), KEGG (https://www.kegg.jp/), NCBI (https://www.ncbi.nlm.nih.gov/), OMIM (https://www.omim.org/), and UniProt (https://www.uniprot.org/), using “Parkinson's Disease” as the keyword, while targets of PD were obtained after deduplication of the results.

### 2.4. Acquisition of Potential Targets against PD

The canonical SMILES of active components were imported into Swiss Target Prediction website (http://www.swisstargetprediction.ch/) to predict targets. The identified PD targets were intersected with the targets of YGJ and YGJ-BBB using the EVenn website tool (http://www.ehbio.com/test/venn/#/) [21]. The intersection targets were considered the potential targets of YGJ and YGJ-BBB against PD.

### 2.5. Construction of Herb-Active Component-Potential Target Network

The active components and potential targets were imported into Cytoscape 3.9.1 software to construct the “Herb-Active component-Potential target” interactive networks, which can show the interconnection between herbs, their corresponding active components, and potential targets [15]. The Analyze Network tool was used for correlation analyses, which can perform topology analyses on each node in the network, store the calculated values as attributes of the corresponding nodes and edges, and can filter network nodes based on the calculated topology metrics. The key components could be selected after sorting the degree value.

### 2.6. Establishment of PPI Network and Enrichment Analyses

The potential targets were input into the STRING website (https://cn.string-db.org/) to construct the protein-protein interaction (PPI) networks. The organism criterion was set as Homo sapiens and the minimum required interaction score was fixed at 0.4. The Molecular Complex Detection (MCODE) algorithm, which can detect densely connected regions that are likely to represent molecular complexes in large PPI networks, was based solely on connectivity data [22]. For the MCODE analyses, the resulting TSV files were downloaded from the STRING database and imported into Cytoscape software, and the MCODE plug-in was used to identify the top 3 clusters of each group.

Afterwards, the Metascape online analysis website (https://metascape.org/gp/ index.html#/main/step1) was used for Gene Ontology (GO) functional and Kyoto Encyclopedia of Genes and Genomes (KEGG) pathway enrichment analyses. Subsequent figure presentation was processed by the OmicStudio tools (https://www.omicstudio.cn/tool/).

### 2.7. Molecular Docking Validation

The potential targets from the KEGG pathway with the highest degree of each group were considered to be the key targets [23]. The procedure of molecular docking was as follows:

1. Target file preparation: The three-dimensional (3D) structures of key targets were retrieved from PDB database (https://www.rcsb.org/) and UniProt website (https://www.uniprot.org/) [24]. Water and ligands of the structure were removed using the PyMOL viewer software, hydrogen bonds were added on the structure by AutoDockTools software, and the structure was saved as a PDBQT file [25]
2. Component file preparation: The 3D structures of key components were downloaded from TCMSP and Pubchem database (https://pubchem.ncbi.nlm.nih.gov/). In AutoDockTools, we input the structure of the component as a ligand and set the structure as follows: delete root, show root expansion, and choose torsions. Then, exported the structure to a ligand file in PDBQT format [26]
3. Constructing grid box: Imported the PDBQT structures of target and ligand into AutoDock4 and defined the grid box of molecular docking. The target structure was used as the grid's center, and the center coordinates (center x/y/z) and box size (size x/y/z) parameters were adjusted to ensure that the grid box completely covers the target structure [27, 28]
4. Molecular docking and visualization: Autogrid program was run for the first docking operation. After Genetic Algorithm was used for the calculating, the Autodock program was run for the second docking operation [29]. The binding energy was calculated and successful docking was defined by a binding energy of ≤ -5 kJ/mol [30]. Figures of the selected binding sites were generated using PyMOL software

## 3. Results

### 3.1. Acquisition of Active Components

79 active components of YGJ were screened out, of which 8 were from Beishashen (BSS), 8 were from Chuanlianzi (CLZ), 2 were from Danggui (DG), 2 were from Dihuang (DH), 42 were from Gouqizi (GQZ), and 26 were from Maidong (MD). The exclusive active components of all 6 herbs are listed in Table 1, and common active components are listed in Table 2. A total of 16 active components that can cross the BBB were identified. To be specific, the number of candidate compounds in BSS, GQZ, and MD was 5, 3, and 8, respectively (Table 3).

### 3.2. Prediction of Potential Targets

**Figure 2:**
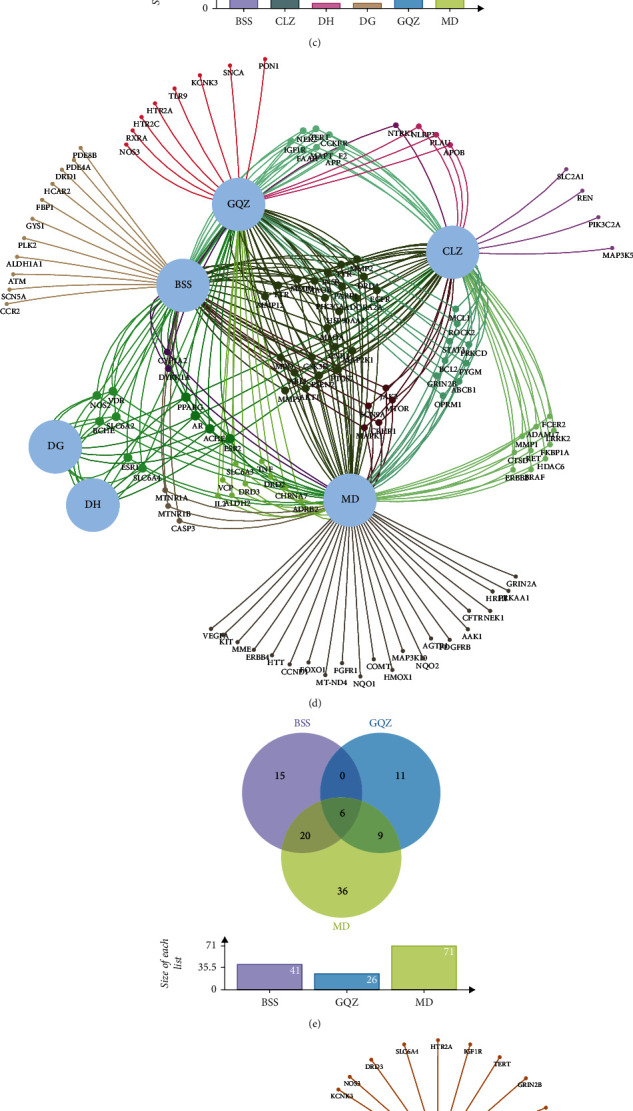
Interactive Venn diagrams. (a) Potential targets of YGJ against PD. (b) Potential targets of YGJ-BBB against PD. (c) Intersections of potential targets of YGJ. (d) Visualization interactive network of potential targets of YGJ. (e) Intersections of potential targets of YGJ-BBB. (f) Visualization interactive network of potential targets of YGJ-BBB.

A total of 728 targets of YGJ, 480 targets of YGJ-BBB, and 836 targets of PD were obtained. After the targets of each group were intersected with the targets of PD, respectively, 128 targets of YGJ (Figure 2(a)) and 97 targets of YGJ-BBB (Figure 2(b)) were considered to be the potential targets against PD. After intersecting the potential targets of each group, the results are shown in Figures 2(c) and 2(d). The results showed that YGJ may synergistically exert the therapeutic effects against PD through multiple potential targets.

### 3.3. Construction and Analyses of Interactive Network

**Figure 3:**
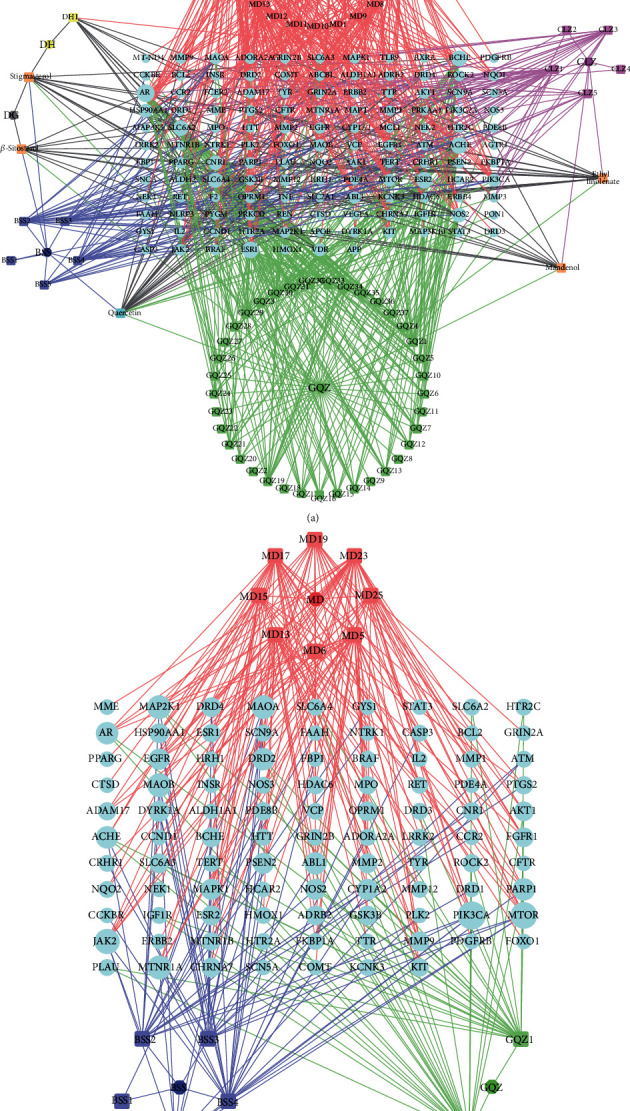
Herb-active component-potential target networks. (a) The network of YGJ. (b) The network of YGJ-BBB.

After the potential targets were obtained, the specific circumstances of the association of each active component with potential targets remain unknown. The “Herb-Active component-Potential target” interactive networks are shown in Figure 3, and 14 active components with degree ≥20 and 12 active components of YGJ-BBB with degree ≥15 were identified to be the key active components [31]; the results are listed in Table 4.

### 3.4. PPI Network Analyses

**Figure 4:**
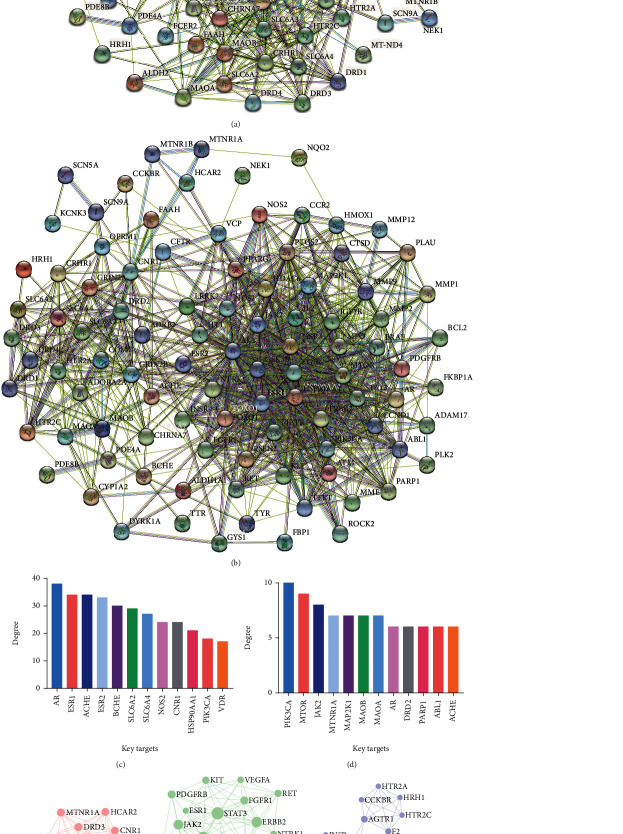
PPI networks and subnetworks analyses. (a) PPI network of potential targets of YGJ. (b) PPI network of potential targets of YGJ-BBB. (c) Bar plot of potential targets of YGJ. The *y*-axis represents the degree of the target. The *x*-axis represents the target. (d) Bar plot of potential targets of YGJ-BBB. The *y*-axis represents the degree of the target. The *x*-axis represents the target. (e) The top 3 clusters of YGJ, identified by MCODE algorithm. (f) The top 3 clusters of YGJ-BBB, identified by MCODE algorithm.

There were interactions between potential targets, and each target involved numerous functional pathways; these were all included in the effective mechanisms. We first established the PPI networks to explore the interactions between potential targets. The PPI network of YGJ consists of 128 nodes and 1398 edges, with an average node degree of 21.8 (Figure 4(a)). The PPI network of YGJ-BBB consists of 97 nodes and 861 edges, with an average node degree of 17.8 (Figure 4(b)). The potential targets with high degree in each group are shown in Figures 4(c) and 4(d), and the degree was listed in Supplementary Materials table S1.

The top 3 MCODE clusters in each group were screened out (Figures 4(e) and 4(f)), and the details of each cluster are listed in Table 5. According to the results, the clusters of YGJ were related to DA synapse, EGFR tyrosine kinase inhibitor resistance, and calcium signaling pathway; the clusters of YGJ-BBB were related to Rap1 signaling pathway, MAPK signaling pathway, and HIF-1 signaling pathway.

### 3.5. Enrichment Analyses

**Figure 5:**
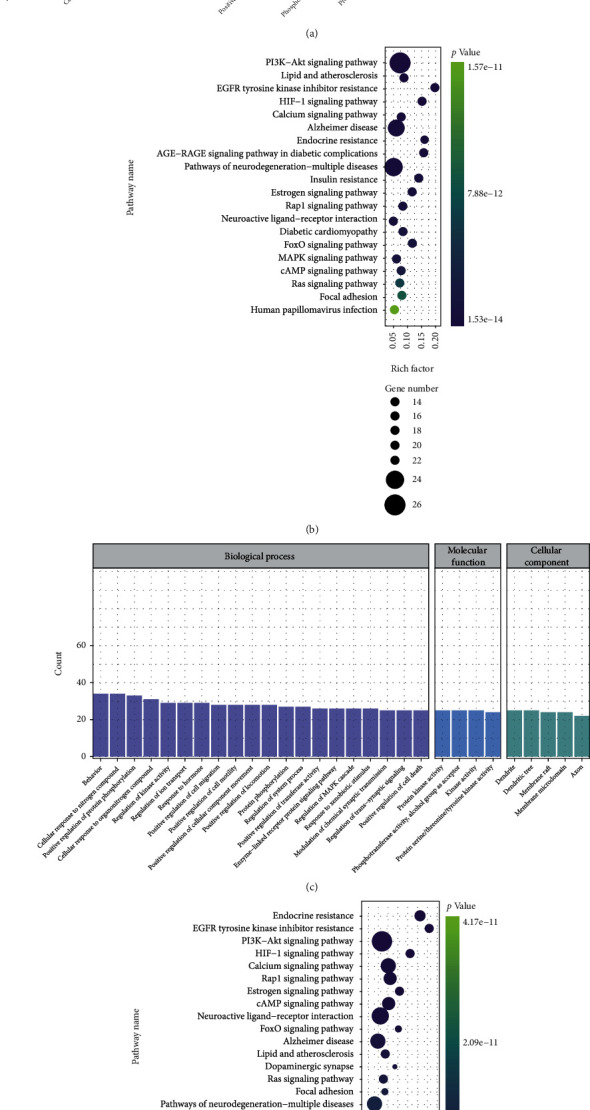
GO functional and KEGG pathway enrichment analyses. (a) GO functional enrichment analyses of YGJ against PD, count over 20. (b) KEGG pathway enrichment analyses of YGJ against PD, sort by *P* value. (c) GO functional enrichment analyses of YGJ-BBB against PD, count over 20. (d) KEGG pathway enrichment analyses of YGJ-BBB against PD, sort by *P* value.

GO items with counts greater than 20 of YGJ are shown in Figure 5(a), with a maximum of 20 items included in each analysis. The biological process (BP) results showed that cellular response to nitrogen compound had the highest count; the molecular function (MF) results were mainly focused on phosphotransferase activity, alcohol group as acceptor; the cellular components (CC) results mainly related to dendrite. Top 20 KEGG pathways of YGJ were sorted by *P* value and shown in Figure 5(b), and the results were mainly concerned in PI3K-Akt signaling pathway. Given the highly distracting capacity of pathways of cancer, relevant cancer pathways were excluded accordingly.

GO items with counts greater than 20 of YGJ-BBB are shown in Figure 5(c), with a maximum of 20 items included in each analysis. The BP results showed that behavior had the highest count; the MF results were mainly focused on protein kinase activity; the CC results mainly related to dendrite. Top 20 KEGG pathways of YGJ-BBB were sorted by *P* value and shown in Figure 5(d), and the results were mainly concerned in endocrine resistance. Relevant cancer pathways were also excluded accordingly.

### 3.6. Molecular Docking Validation

**Figure 6:**
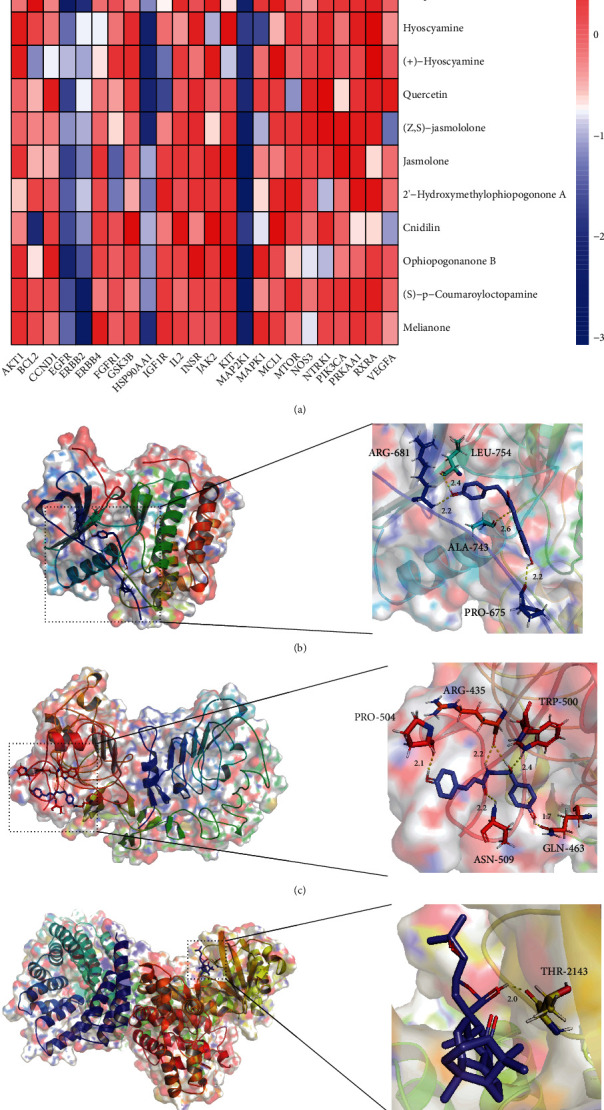
Heatmap and representative molecular docking results of YGJ. (a) Heatmap of molecular docking results of YGJ. (b) Schematic diagram of N-coumaroyltyramine-EGFR. (c) Schematic diagram of (S)-p-coumaroyloctopamine-ERBB2. (d) Schematic diagram of melianone-MTOR.

**Figure 7:**
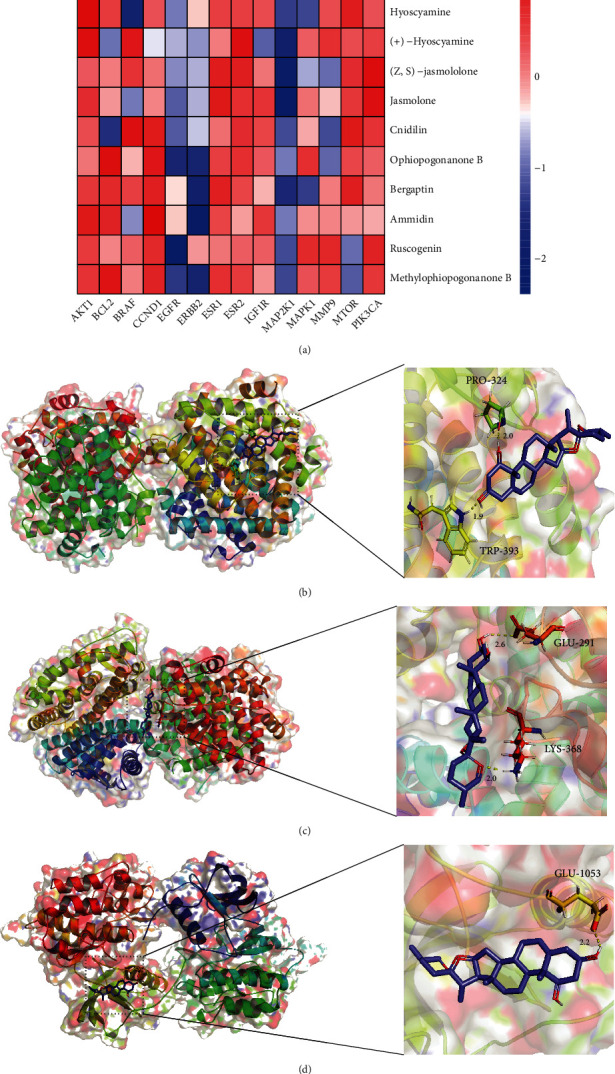
Heatmap and representative molecular docking results of YGJ-BBB. (a) Heatmap of molecular docking results of YGJ-BBB. (b) Schematic diagram of ruscogenin-ESR1. (c) Schematic diagram of ruscogenin-ESR2. (d) Schematic diagram of ruscogenin-IGF1R.

The key targets are listed in Table 6, and the PDB IDs were listed in Supplementary Materials table S2. The key targets were docked crossly with the key components of each group to predict the effective mechanisms. The successful docking results are listed in Table 7, and 3 representative successful results of each group were selected out to form schematic diagrams (Figures 6 and 7). Full results were listed in Supplementary Materials table S3. According to the representative successful results, orchinol can be docked with ERBB2 target, N-coumaroyltyramine can be docked with EGFR target, melianone can be docked with MTOR target, and ruscogenin can be docked with ESR1, ESR2, and IGF1R targets.

## 4. Discussion

Neuroinflammation plays a significant role in PD etiology along with mitochondrial dysfunction and impaired proteostasis. Proinflammatory factors secreted by senescent cells in the brain trigger neuroinflammation, leading to immune cell-mediated apoptosis of DA neurons. Neurodegeneration and neuroinflammation would feed each other and promote disease progression [32–34]. Oxidative stress can not only participate in the formation of amyloid by affecting the structure and self-assembly of *α*-syn but also be interrelated and interdependent with the inflammatory process [35]. Multiple causes of mitochondrial damage-mediated apoptosis of DA neurons are also associated with the development of PD [36]. Therefore, inhibition of the inflammatory response, oxidative stress, and apoptosis of neuronal cells through systemic effects of the body or interventions targeting the brain has significant therapeutic effects on PD.

Multiple active components screened in this study have been confirmed to be able to exert therapeutic effects on PD through the above-mentioned approaches. Among the active components crossing the BBB, isoimperatorin can inhibit the nuclear factor (NF)-*κ*B pathway, reduce the levels of interleukin (IL)-4, IL-5, IL-6, IL-13, and tumor necrosis factor (TNF)-*α*, and possess the anti-inflammation effect [37]. Methylophiopogonanone B has antioxidant property, can inhibit the production of malondialdehyde (MDA) and reactive oxygen species (ROS), enhance the activity of superoxide dismutase (SOD), and down-regulate the expressions of Bax/Bcl-2 and caspase-3 to inhibit apoptosis, while can also significantly down-regulate the expression of IL-6 and IL-8 [38]. Ruscogenin can inhibit the activity of the NF-*κ*B pathway to reduce the expression of inflammatory cytokines, including IL-1*β* and caspase-1, and also reduce the production of ROS, making it both anti-inflammatory and antioxidant properties [39]. Thus, active components crossing the BBB can inhibit inflammatory responses, oxidative stress, and neuronal apoptosis to treat PD.

The MCODE algorithm can detect densely connected regions likely to represent molecular complexes in large PPI networks, based solely on connectivity data [40]. The top 3 clusters of YGJ were related to DA synapse, EGFR tyrosine kinase inhibitor resistance, and calcium signaling pathway. Many disorders with control deficits are associated with abnormal dopamine transmission [24]. Improvement of DA transmission abnormality and elevation of DA level may contribute to rapid reversal of motor complications of PD [41, 42]. Epidermal growth factor receptor (EGFR) is a tyrosine kinase receptor involved in cell differentiation and proliferation, and its mutation and amplification are associated with the pathology of neurodegenerative diseases [43, 44]. Amyloid can be transported between neuronal cells through EGFR-mediated endocytosis, thereby enabling amyloid transfer throughout the brain [45, 46]. Calcium can bind to the C-terminus of *α*-syn and promote the secretion and aggregation of *α*-syn [47, 48]. Activation of the calcium signaling pathway can promote the generation of mitochondrial oxidative stress and the apoptosis of substantia nigra DA neurons [49, 50]. Therefore, YGJ can improve the transmission abnormality of DA and amyloid and inhibit the secretion and aggregation of *α*-syn through systemic effects. The top 3 clusters of YGJ-BBB were related to Rap1 signaling pathway, MAPK signaling pathway, and HIF-1 signaling pathway. Activation of the Ras-association proximate 1 (Rap1) and mitogen-activated protein kinase (MAPK) signaling pathways has been shown to be able to promote inflammatory responses and ROS production [51–54], while activation of the hypoxia-inducible factor-1 (HIF-1) signaling pathway could inhibit the mitochondria-mediated apoptotic process [55]. The MAPK pathway can be activated by its upstream Rap1 pathway, which in turn activates the downstream HIF-1 pathway [56]. Therefore, down-regulation of Rap1, MAPK, and HIF-1 pathways can reduce neuronal cell apoptosis by inhibiting inflammatory responses, oxidative stress, and mitochondrial damage in the brain.

The efficacy of TCM is represented by the synergy effects of potential targets, but experimental validation would yield massive work and thus hinder our progression when we scrutinized the TCM-targets effects relationship spectrum. KEGG enrichment analysis can find significant signal pathways for the synergy effects of potential targets. Therefore, we need to step-wisely narrow our study objects through KEGG enrichment analysis. According to the results of the KEGG pathway enrichment analyses, YGJ group was mainly focused on PI3K-Akt signaling pathway, while YGJ-BBB group was mainly concerned in endocrine resistance. Activating the PI3K-Akt cell survival pathway can inhibit mitochondrial damage-mediated apoptosis, promote autophagy of amyloid, and play a neuroprotective role [57–59]. In the meantime, the activation of the PI3K-Akt pathway can also inhibit inflammation response and oxidative stress [60, 61]. EGFR and ERBB2 are upstream ligands [62, 63], while CCND1, MTOR, and VEGFA are downstream receptors of PI3K-Akt signaling pathway [64–66], and interventions on targets above-mentioned can all have effects on this pathway. The MAPK pathway and the PI3K-Akt pathway have extensive interactions, and the PI3K-Akt pathway can be regulated by acting on the important targets of the MAPK pathway, MAP2K1 and MAPK1 [67, 68]. According to the docking results, it is speculated that YGJ may act on CCND1, EGFR, ERBB2, MAP2K1, MAPK1, MTOR, and VEGFA targets to modulate PI3K-Akt signaling pathway, thereby treating PD by affecting the peripheral system.

Studies have shown that the regulation of the endocrine system also plays an important role in the treatment of PD, including sex hormones, insulin, and melatonin [69–72]. According to the docking results, it is speculated that YGJ-BBB may act on ERBB2, ESR1, ESR2, IGF1R, MAP2K1, and MAPK1 targets to intervene endocrine resistance. Estrogen has been shown to increase the synthesis, release, re-uptake, and turnover of DA [73], prevent memory impairment by inhibiting NF-*κ*B activity to modulate neurogenic inflammation [74], and improve mitochondrial damage and restore the activity of antioxidant enzymes [75], thereby reducing the risk of PD and improving symptoms of disorders with control deficits [76, 77]. The docking results showed that YGJ-BBB can act on estrogen receptor 1 (ESR1) and estrogen receptor 2 (ESR2), improve the utilization rate of estrogen, and promote the therapeutic effect on PD. There is accumulating evidence that insulin can cross the BBB and influence a multitude of processes in the brain, including modulation of neuronal survival and growth [78], DA transmission [79], maintenance of synapses [80], autophagy of amyloid [81], oxidative stress [82], and neuroinflammation [83]. Meanwhile, a process analogous to insulin resistance exists in the brains of PD patients. Therefore, improving the utilization and restoring the normal function of insulin in the brain could be used as a strategy to slow the progression of PD. The docking results showed that YGJ-BBB can act on insulin-like growth factor 1 receptor (IGF1R) to achieve this. In addition, MAPK signaling pathway is the classical pathway that can be activated by estrogen and insulin. Intervention of the important targets MAP2K1 and MAPK1 in this pathway and the upstream pathway target ERBB2 can affect the function of estrogen and insulin. That is to say, the active components of YGJ that cross the BBB can restore the normal function of estrogen and insulin in the brain by acting on ERBB2, ESR1, ESR2, IGF1R, MAP2K1, and MAPK1, thereby reducing the incidence, slowing down the progression, and improving related symptoms of PD by affecting the central nervous. It is worth noting that the expression of PI3K-Akt signaling pathway is also closely related to the effects of estrogen and insulin [84–86], which indicates that the active components of YGJ can also enhance the therapeutic effects of the active components that cross the BBB on central nervous through peripheral system effects triggered by PI3K-Akt pathway.

Despite the encouraging discoveries, there still exist certain limitations. To begin with, our study is largely constructed upon current databases. However, scientific studies regarding TCM against PD remain insufficient compared to other diseases and thus require further attention. This can be reflected by the KEGG enrichment analyses in which the overlay between disease pathways could induce inadequate inference. Moreover, the comprehensive compounds of herbs in the YGJ are still missing. Those unidentified and unrecorded components were not included and might affect the present result. Although we take a step forward in systemic explanation regarding TCM mechanism from central nervous (inner brain) and peripheral system (outer brain), the determined bioactive component and their corresponding targets cannot fully represent the holistic concept of YGJ. Since the efficacy of YGJ has been clinically testified, we believe that the further advance in computational techniques combined with experimental validation can benefit the exploration of underlying mechanism on YGJ against PD.

## 5. Conclusion

In conclusion, 79 active components were screened in this study, of which 16 active components can permeate the BBB. Overall, we identified 128 potential targets of YGJ, 97 potential targets of YGJ-BBB potentially suitable for the treatment of PD. The herb-active component-potential target network, PPI networks, and MCODE networks were constructed through the approach of network pharmacology. It is speculated that orchinol, N-coumaroyltyramine, (S)-p-coumaroyloctopamine, and melianone can bind with the targets related to PI3K-Akt and its upstream and downstream pathways to treat PD by affecting the peripheral system. And bergaptin, ammidin, and ruscogenin, the active components of YGJ that cross the BBB, can bind with targets related to the regulation of multiple hormones to modulate endocrine resistance, such as restoring the normal function of estrogen and insulin to exert therapeutic effects on the central nervous, which can also be enhanced through peripheral system effects of PI3K-Akt pathway. Therefore, not only YGJ can have different therapeutic effects on PD through the effects of peripheral system and central nervous but also the two approaches can work in coordination, thus reflecting the systematic collaboration and single-targetedness of YGJ against PD.

## Figures and Tables

**Table 1:** Exclusive active components of all 6 herbs of YGJ.

Herb	No.	Active component
BSS	BSS1	Alloisoimperatorin
	BSS2	Ammidin
	BSS3	Bergaptin
	BSS4	Cnidilin
	BSS5	Isoimperatorin
CLZ	CLZ1	(E)-3-[(2S,3R)-2-(4-hydroxy-3-methoxy-phenyl)-7-methoxy-3-methylol-2,3-dihydrobenzofuran-5-yl]acrolein
	CLZ2	Medioresinol
	CLZ3	Melianone
	CLZ4	Nimbolidin D
	CLZ5	Nimbolin A
DH	DH1	Sitosterol
GQZ	GQZ1	(+)-Hyoscyamine
	GQZ2	(24R)-4alpha-Methyl-24-ethylcholesta-7,25-dien-3beta-ylacetate
	GQZ3	(E,E)-1-ethyl octadeca-3,13-dienoate
	GQZ4	14b-pregnane
	GQZ5	24-ethylcholest-22-enol
	GQZ6	24-ethylcholesta-5,22-dienol
	GQZ7	24-methyl-31-norlanost-9(11)-enol
	GQZ8	24-Methylenecycloartan-3beta,21-diol
	GQZ9	24-methylenelanost-8-enol
	GQZ10	24-methylidenelophenol
	GQZ11	31-Norcyclolaudenol
	GQZ12	31-norlanost-9(11)-enol
	GQZ13	31-norlanosterol
	GQZ14	4,24-methyllophenol
	GQZ15	4alpha,14alpha,24-trimethylcholesta-8,24-dienol
	GQZ16	4alpha,24-dimethylcholesta-7,24-dienol
	GQZ17	4alpha-methyl-24-ethylcholesta-7,24-dienol
	GQZ18	6-Fluoroindole-7-Dehydrocholesterol
	GQZ19	7-O-Methylluteolin-6-C-beta-glucoside_qt
	GQZ20	Campesterol
	GQZ21	CLR
	GQZ22	Cryptoxanthin monoepoxide
	GQZ23	Cyanin
	GQZ24	Cycloartenol
	GQZ25	Cycloeucalenol
	GQZ26	Daucosterol_qt
	GQZ27	*δ*-Carotene
	GQZ28	Fucosterol
	GQZ29	Glycitein
	GQZ30	Hyoscyamine
	GQZ31	LAN
	GQZ32	Lanost-8-en-3beta-ol
	GQZ33	Lanost-8-enol
	GQZ34	Lantadene A
	GQZ35	Lophenol
	GQZ36	Obtusifoliol
	GQZ37	Sitosterol alpha1
MD	MD1	(1S,2R,4S)-Borneol beta-D-glucopyranoside
	MD2	(3R)-5,7-dihydroxy-3-[(2-hydroxy-4-methoxyphenyl)methyl]-8-methoxy-6-methyl-2,3-dihydrochromen-4-one
	MD3	(3R)-5,7-dihydroxy-3-[(4-hydroxy-3-methoxyphenyl)methyl]-6,8-dimethyl-2,3-dihydrochromen-4-one
	MD4	(S)-p-Coumaroyloctopamine
	MD5	(Z,S)-Jasmololone
	MD6	2-Ethylhexyl 3-aminopropyl ether
	MD7	2′-Hydroxymethylophiopogonone A
	MD8	5,7,2′-Trihydroxy-6-Methyl-3-(3′,4′-Methylenedioxybenzyl) Chromone
	MD9	5,7-Dihydroxy-3-[(4-methoxyphenyl)methyl]-8-methyl-4-oxochromene-6-carbaldehyde
	MD10	6-Aldehydo-isoophiopogonone A
	MD11	Cyperene
	MD12	DL-threo-beta-Hydroxyaspartic acid
	MD13	Jasmolone
	MD14	Methylophiopogonanone A
	MD15	Methylophiopogonanone B
	MD16	Moupinamide
	MD17	N-coumaroyltyramine
	MD18	Ophiopogonanone A
	MD19	Ophiopogonanone B
	MD20	Ophiopogonanone C
	MD21	Ophiopogonone A
	MD22	Ophiopogonone C
	MD23	Orchinol
	MD24	Poriferasterol
	MD25	Ruscogenin
	MD26	Ruscogenin 1-O-Sulfate

**Table 2:** Common active components of YGJ.

Active component	Herbs
*β*-Sitosterol	BSS, DG, GQZ
Ethyl linolenate	CLZ, GQZ
Mandenol	CLZ, GQZ
Quercetin	BSS, CLZ, GQZ
Stigmasterol	BSS, DG, DH, GQZ

**Table 3:** Active components crossing the BBB.

Herb	No.	Active component
BSS	1	Alloisoimperatorin
	2	Ammidin
	3	Bergaptin
	4	Cnidilin
	5	Isoimperatorin

GQZ	1	(+)-Hyoscyamine
	2	Cyanin
	3	Hyoscyamine

MD	1	(Z,S)-Jasmololone
	2	2-Ethylhexyl 3-aminopropyl ether
	3	Jasmolone
	4	Methylophiopogonanone B
	5	N-coumaroyltyramine
	6	Ophiopogonanone B
	7	Orchinol
	8	Ruscogenin

**Table 4:** The degree of key components of YGJ and YGJ-BBB.

Group	No.	Key component	Degree
YGJ	1	Orchinol	32
	2	N-coumaroyltyramine	28
	3	(E)-3-[(2S,3R)-2-(4-hydroxy-3-methoxy-phenyl)-7-methoxy-3-methylol-2,3-dihydrobenzofuran-5-yl]acrolein	28
	4	Moupinamide	27
	5	Hyoscyamine	27
	6	(+)-Hyoscyamine	27
	7	Quercetin	27
	8	(Z,S)-Jasmololone	24
	9	Jasmolone	24
	10	2′-Hydroxymethylophiopogonone A	23
	11	Cnidilin	23
	12	Ophiopogonanone B	22
	13	(S)-p-Coumaroyloctopamine	21
	14	Melianone	21

YGJ-BBB	1	Orchinol	32
	2	N-coumaroyltyramine	28
	3	Hyoscyamine	27
	4	(+)-Hyoscyamine	27
	5	(Z,S)-Jasmololone	24
	6	Jasmolone	24
	7	Cnidilin	23
	8	Ophiopogonanone B	22
	9	Bergaptin	20
	10	Ammidin	19
	11	Ruscogenin	17
	12	Methylophiopogonanone B	17

**Table 5:** Top 3 clusters identified, respectively, from MCODE results of YGJ and YGJ-BBB.

Group	Cluster	Pathway description	Targets	Count	Score
YGJ	1	Dopaminergic synapse	APP, DRD4, HCAR2, CCR2, MTNR1B, DRD3, OPRM1, MTNR1A, DRD2, CNR1	10	4.50
	2	EGFR tyrosine kinase inhibitor resistance	PDGFRB, KIT, MAPT, LRRK2, MAP2K1, PRKCD, NTRK1, PIK3CA, ERBB2, RET, JAK2, VEGFA, ESR1, MTOR, IGF1R, STAT3, BRAF, IL2, GSK3B, FGFR1, HDAC6	21	4.48
	3	Calcium signaling pathway	ESR2, HTR2A, INSR, NOS2, HTR2C, MCL1, VCP, ERBB4, SNCA, AGTR1, F2, HRH1, TERT, ROCK2, EGFR, HSP90AA1, MAPK1, CCKBR	18	2.61

YGJ-BBB	1	Rap1 signaling pathway	DRD3, PDGFRB, MTNR1B, CCR2, DRD4, HCAR2, DRD2, MTNR1A, OPRM1, KIT, CNR1, IL2, MAP2K1, JAK2	14	3.14
	2	MAPK signaling pathway	NTRK1, ESR1, EGFR, STAT3, ERBB2, IGF1R	6	2.33
	3	HIF-1 signaling pathway	MTOR, BRAF, MAPK1, ROCK2, INSR, PIK3CA, NOS2, HSP90AA1, TERT, FGFR1	10	2.10

**Table 6:** Key targets of YGJ and YGJ-BBB.

Group	KEGG pathway	Key targets
YGJ	PI3K-Akt signaling pathway	AKT1, CCND1, BCL2, EGFR, ERBB2, ERBB4, FGFR1, MTOR, GSK3B, GYS1, HSP90AA1, IGF1R, IL2, INSR, JAK2, KIT, MCL1, NOS3, NTRK1, PDGFRB, PIK3CA, PRKAA1, MAPK1, MAP2K1, RXRA, VEGFA
YGJ-BBB	Endocrine resistance	AKT1, CCND1, BCL2, BRAF, EGFR, ERBB2, ESR1, ESR2, MTOR, IGF1R, MMP2, MMP9, PIK3CA, MAPK1, MAP2K1

**Table 7:** Basic information of representative molecular docking results.

Group	Active component	Target	Residue	Energy (kJ/mol)	Docking distance (Å)
YGJ	Orchinol	ERBB2	ARG-182	-5.16	1.80
	N-coumaroyltyramine	EGFR	ARG-681	-5.25	2.20
			LEU-754		2.40
			ALA-743		2.60
			PRO-675		2.20
	N-coumaroyltyramine	MAP2K1	ASN-319	-5.14	2.10
			PRO-323		2.20
			LYS-344		2.20
	N-coumaroyltyramine	MAPK1	ASP-100	-5.09	2.00
			LYS-99		2.20
			ALA-9		2.20
	(S)-p-Coumaroyloctopamine	ERBB2	GLN-463	-5.53	1.70
			PRO-504		2.10
			ASN-509		2.20
			ARG-435		2.20
			TRP-500		2.40
	(S)-p-Coumaroyloctopamine	MAP2K1	ASP-190	-5.93	2.30
	Melianone	CCND1	THR-80	-5.11	2.10
			ARG-5		2.30
	Melianone	MTOR	THR-2143	-5.19	2.00
	Melianone	VEGFA	CYS-61	-5.29	2.10

YGJ-BBB	Bergaptin	ERBB2	GLN-308	-5.78	2.00
	Bergaptin	MAP2K1	LEU-63	-5.55	2.10
	Bergaptin	MAPK1	LYS-138	-5.33	1.80
	Ammidin	ERBB2	GLN-299	-5.93	2.00
			TYR-322		2.20
	Ammidin	MAP2K1	LYS-97	-5.06	2.20
	Ruscogenin	ESR1	TRP-393	-5.49	1.90
			PRO-324		2.00
	Ruscogenin	ESR2	LYS-368	-5.28	2.00
			GLU-291		2.60
	Ruscogenin	IGF1R	GLU-1053	-5.32	2.20

## Data Availability

The data used to support the findings of this study are included within the article and supplementary materials.

## Supplementary Materials

Supplementary Materials Table S1. Degree of the potential targets of YGJ and YGJ-BBB. Table S2. PDB ID of key targets. Table S3. Basic information of all molecular docking results.
