# Supplementary material for: Network Pharmacology and Molecular Docking Analyses Unveil the Mechanisms of Yiguanjian Decoction against Parkinson's Disease from Inner/Outer Brain Perspective
Source: Biomed Res Int. 2022 Sep 26;2022:4758189. doi: 10.1155/2022/4758189 (PMC9552692; doi:10.1155/2022/4758189)
Supplement: Supplementary Materials — Table S1. Degree of the potential targets of YGJ and YGJ-BBB. Table S2. PDB ID of key targets. Table S3. Basic information of all molecular docking results. [file 4758189.f1.docx]

### ***Supplementary Materials***

### **Network pharmacology and molecular docking analyses unveil the mechanisms of Yiguanjian Decoction against Parkinson's Disease from inner/outer brain perspectives**

Zhongqi Shen ^1^, Meng Yu ^2, *^, Shaozhi Zhang ^2, *^

**Affiliations**

^1^ Institute of Traditional Chinese Medicine Innovative Research, Shandong University of Traditional Chinese Medicine, Jinan, Shandong, P. R. China 250355.

^2^ College of Traditional Chinese Medicine, Shandong University of Traditional Chinese Medicine, Jinan, Shandong, P. R. China 250355.

^*^ Corresponding authors. E-mail addresses: 60230037@sdutcm.edu.cn (M. Yu), and 60230073@sdutcm.edu.cn (S. Z. Zhang).

### **Supplementary Materials**

Table S1. Degree of the potential targets of YGJ and YGJ-BBB.

Table S2. PDB ID of key targets.

Table S3. Basic information of all molecular docking results.

***Supplementary Tables***

| **Supplementary Table S1. Degree of the potential targets of YGJ and YGJ-BBB.** | | | |
| --- | --- | --- | --- |
| Group | No. | Potential target | Degree |
| YGJ | 1 | AR | 38 |
|  | 2 | ESR1 | 34 |
|  | 3 | ACHE | 34 |
|  | 4 | ESR2 | 33 |
|  | 5 | BCHE | 30 |
|  | 6 | SLC6A2 | 29 |
|  | 7 | SLC6A4 | 27 |
|  | 8 | NOS2 | 24 |
|  | 9 | CNR1 | 24 |
|  | 10 | HSP90AA1 | 21 |
|  | 11 | PIK3CA | 18 |
|  | 12 | VDR | 17 |
| YGJ-BBB | 1 | PIK3CA | 10 |
|  | 2 | MTOR | 9 |
|  | 3 | JAK2 | 8 |
|  | 4 | MTNR1A | 7 |
|  | 5 | MAP2K1 | 7 |
|  | 6 | MAOB | 7 |
|  | 7 | MAOA | 7 |
|  | 8 | AR | 6 |
|  | 9 | DRD2 | 6 |
|  | 10 | PARP1 | 6 |
|  | 11 | ABL1 | 6 |
|  | 12 | ACHE | 6 |

| **Supplementary Table S2. PDB ID of key targets.** | |
| --- | --- |
| Key target | PDB ID |
| AKT1 | 3O96 |
| BCL2 | 2XA0 |
| BRAF | 1UWH |
| CCND1 | 2W9F |
| EGFR | 2GS2 |
| ERBB2 | 3PP0 |
| ERBB4 | 3BBT |
| ESR1 | 3OS8 |
| ESR2 | 1U3Q |
| FGFR1 | 1FGI |
| GSK3B | 1H8F |
| HSP90AA1 | 1UYH |
| IGF1R | 1M7N |
| IL2 | 1QVN |
| INSR | 1I44 |
| JAK2 | 2B7A |
| KIT | 1PKG |
| MAP2K1 | 2P55 |
| MAPK1 | 1TVO |
| MCL1 | 4HW2 |
| MMP2 | 7XGJ |
| MMP9 | 1L6J |
| MTOR | 3JBZ |
| NOS3 | 1M9R |
| NTRK1 | 4PMS |
| PDGFRB | 1GQ5 |
| PIK3CA | 3I5S |
| PRKAA1 | 4RED |
| RXRA | 1MV9 |
| VEGFA | 1MKG |

| **Supplementary Table S3. Basic information of all molecular docking results.** | | | |
| --- | --- | --- | --- |
| Group | Active component | Target | Docking energy (kJ/mol) |
| YGJ | Orchinol | AKT1 | -3.74 |
|  | Orchinol | BCL2 | -3.21 |
|  | Orchinol | CCND1 | -3.62 |
|  | Orchinol | EGFR | -4.08 |
|  | Orchinol | ERBB2 | -5.16 |
|  | Orchinol | ERBB4 | -3.31 |
|  | Orchinol | FGFR1 | -4.22 |
|  | Orchinol | GSK3B | -4.28 |
|  | Orchinol | HSP90AA1 | -4.58 |
|  | Orchinol | IGF1R | -3.72 |
|  | Orchinol | IL2 | -3.74 |
|  | Orchinol | INSR | -2.91 |
|  | Orchinol | JAK2 | -3.59 |
|  | Orchinol | KIT | -3.17 |
|  | Orchinol | MAP2K1 | -4.56 |
|  | Orchinol | MAPK1 | -3.25 |
|  | Orchinol | MCL1 | -3.26 |
|  | Orchinol | MTOR | -3.47 |
|  | Orchinol | NOS3 | -3.26 |
|  | Orchinol | NTRK1 | -3.88 |
|  | Orchinol | PIK3CA | -3.71 |
|  | Orchinol | PRKAA1 | -3.06 |
|  | Orchinol | RXRA | -3.03 |
|  | Orchinol | VEGFA | -4.42 |
|  | N-coumaroyltyramine | AKT1 | -2.92 |
|  | N-coumaroyltyramine | BCL2 | -3.80 |
|  | N-coumaroyltyramine | CCND1 | -2.80 |
|  | N-coumaroyltyramine | EGFR | -5.25 |
|  | N-coumaroyltyramine | ERBB2 | -4.59 |
|  | N-coumaroyltyramine | ERBB4 | -3.21 |
|  | N-coumaroyltyramine | FGFR1 | -2.98 |
|  | N-coumaroyltyramine | GSK3B | -3.32 |
|  | N-coumaroyltyramine | HSP90AA1 | -4.58 |
|  | N-coumaroyltyramine | IGF1R | -3.42 |
|  | N-coumaroyltyramine | IL2 | -3.16 |
|  | N-coumaroyltyramine | INSR | -2.60 |
|  | N-coumaroyltyramine | JAK2 | -2.29 |
|  | N-coumaroyltyramine | KIT | -2.92 |
|  | N-coumaroyltyramine | MAP2K1 | -5.14 |
|  | N-coumaroyltyramine | MAPK1 | -5.09 |
|  | N-coumaroyltyramine | MCL1 | -2.73 |
|  | N-coumaroyltyramine | MTOR | -3.33 |
|  | N-coumaroyltyramine | NOS3 | -3.30 |
|  | N-coumaroyltyramine | NTRK1 | -2.42 |
|  | N-coumaroyltyramine | PIK3CA | -2.92 |
|  | N-coumaroyltyramine | PRKAA1 | -3.19 |
|  | N-coumaroyltyramine | RXRA | -2.48 |
|  | N-coumaroyltyramine | VEGFA | -2.90 |
|  | (E)-3-[(2S,3R)-2-(4-hydroxy-3-methoxy-phenyl)-7-methoxy-3-methylol-2,3-dihydrobenzofuran-5-yl]acrolein | AKT1 | -1.72 |
|  | (E)-3-[(2S,3R)-2-(4-hydroxy-3-methoxy-phenyl)-7-methoxy-3-methylol-2,3-dihydrobenzofuran-5-yl]acrolein | BCL2 | -1.79 |
|  | (E)-3-[(2S,3R)-2-(4-hydroxy-3-methoxy-phenyl)-7-methoxy-3-methylol-2,3-dihydrobenzofuran-5-yl]acrolein | CCND1 | -2.31 |
|  | (E)-3-[(2S,3R)-2-(4-hydroxy-3-methoxy-phenyl)-7-methoxy-3-methylol-2,3-dihydrobenzofuran-5-yl]acrolein | EGFR | -3.74 |
|  | (E)-3-[(2S,3R)-2-(4-hydroxy-3-methoxy-phenyl)-7-methoxy-3-methylol-2,3-dihydrobenzofuran-5-yl]acrolein | ERBB2 | -4.29 |
|  | (E)-3-[(2S,3R)-2-(4-hydroxy-3-methoxy-phenyl)-7-methoxy-3-methylol-2,3-dihydrobenzofuran-5-yl]acrolein | ERBB4 | -2.88 |
|  | (E)-3-[(2S,3R)-2-(4-hydroxy-3-methoxy-phenyl)-7-methoxy-3-methylol-2,3-dihydrobenzofuran-5-yl]acrolein | FGFR1 | -2.41 |
|  | (E)-3-[(2S,3R)-2-(4-hydroxy-3-methoxy-phenyl)-7-methoxy-3-methylol-2,3-dihydrobenzofuran-5-yl]acrolein | GSK3B | -2.36 |
|  | (E)-3-[(2S,3R)-2-(4-hydroxy-3-methoxy-phenyl)-7-methoxy-3-methylol-2,3-dihydrobenzofuran-5-yl]acrolein | HSP90AA1 | -2.97 |
|  | (E)-3-[(2S,3R)-2-(4-hydroxy-3-methoxy-phenyl)-7-methoxy-3-methylol-2,3-dihydrobenzofuran-5-yl]acrolein | IGF1R | -2.10 |
|  | (E)-3-[(2S,3R)-2-(4-hydroxy-3-methoxy-phenyl)-7-methoxy-3-methylol-2,3-dihydrobenzofuran-5-yl]acrolein | IL2 | -3.57 |
|  | (E)-3-[(2S,3R)-2-(4-hydroxy-3-methoxy-phenyl)-7-methoxy-3-methylol-2,3-dihydrobenzofuran-5-yl]acrolein | INSR | -1.98 |
|  | (E)-3-[(2S,3R)-2-(4-hydroxy-3-methoxy-phenyl)-7-methoxy-3-methylol-2,3-dihydrobenzofuran-5-yl]acrolein | JAK2 | -3.30 |
|  | (E)-3-[(2S,3R)-2-(4-hydroxy-3-methoxy-phenyl)-7-methoxy-3-methylol-2,3-dihydrobenzofuran-5-yl]acrolein | KIT | -2.78 |
|  | (E)-3-[(2S,3R)-2-(4-hydroxy-3-methoxy-phenyl)-7-methoxy-3-methylol-2,3-dihydrobenzofuran-5-yl]acrolein | MAP2K1 | -4.69 |
|  | (E)-3-[(2S,3R)-2-(4-hydroxy-3-methoxy-phenyl)-7-methoxy-3-methylol-2,3-dihydrobenzofuran-5-yl]acrolein | MAPK1 | -2.17 |
|  | (E)-3-[(2S,3R)-2-(4-hydroxy-3-methoxy-phenyl)-7-methoxy-3-methylol-2,3-dihydrobenzofuran-5-yl]acrolein | MCL1 | -2.29 |
|  | (E)-3-[(2S,3R)-2-(4-hydroxy-3-methoxy-phenyl)-7-methoxy-3-methylol-2,3-dihydrobenzofuran-5-yl]acrolein | MTOR | -2.64 |
|  | (E)-3-[(2S,3R)-2-(4-hydroxy-3-methoxy-phenyl)-7-methoxy-3-methylol-2,3-dihydrobenzofuran-5-yl]acrolein | NOS3 | -2.17 |
|  | (E)-3-[(2S,3R)-2-(4-hydroxy-3-methoxy-phenyl)-7-methoxy-3-methylol-2,3-dihydrobenzofuran-5-yl]acrolein | NTRK1 | -2.99 |
|  | (E)-3-[(2S,3R)-2-(4-hydroxy-3-methoxy-phenyl)-7-methoxy-3-methylol-2,3-dihydrobenzofuran-5-yl]acrolein | PIK3CA | -2.92 |
|  | (E)-3-[(2S,3R)-2-(4-hydroxy-3-methoxy-phenyl)-7-methoxy-3-methylol-2,3-dihydrobenzofuran-5-yl]acrolein | PRKAA1 | -2.01 |
|  | (E)-3-[(2S,3R)-2-(4-hydroxy-3-methoxy-phenyl)-7-methoxy-3-methylol-2,3-dihydrobenzofuran-5-yl]acrolein | RXRA | -2.36 |
|  | (E)-3-[(2S,3R)-2-(4-hydroxy-3-methoxy-phenyl)-7-methoxy-3-methylol-2,3-dihydrobenzofuran-5-yl]acrolein | VEGFA | -2.85 |
|  | Moupinamide | AKT1 | -2.96 |
|  | Moupinamide | BCL2 | -2.10 |
|  | Moupinamide | CCND1 | -3.05 |
|  | Moupinamide | EGFR | -4.60 |
|  | Moupinamide | ERBB2 | -4.26 |
|  | Moupinamide | ERBB4 | -2.95 |
|  | Moupinamide | FGFR1 | -2.76 |
|  | Moupinamide | GSK3B | -3.01 |
|  | Moupinamide | HSP90AA1 | -3.68 |
|  | Moupinamide | IGF1R | -3.37 |
|  | Moupinamide | IL2 | -2.45 |
|  | Moupinamide | INSR | -2.77 |
|  | Moupinamide | JAK2 | -2.31 |
|  | Moupinamide | KIT | -3.32 |
|  | Moupinamide | MAP2K1 | -4.49 |
|  | Moupinamide | MAPK1 | -2.57 |
|  | Moupinamide | MCL1 | -2.26 |
|  | Moupinamide | MTOR | -2.92 |
|  | Moupinamide | NOS3 | -3.09 |
|  | Moupinamide | NTRK1 | -2.57 |
|  | Moupinamide | PIK3CA | -2.57 |
|  | Moupinamide | PRKAA1 | -2.51 |
|  | Moupinamide | RXRA | -2.38 |
|  | Moupinamide | VEGFA | -2.86 |
|  | Hyoscyamine | AKT1 | -2.51 |
|  | Hyoscyamine | BCL2 | -2.95 |
|  | Hyoscyamine | CCND1 | -3.23 |
|  | Hyoscyamine | EGFR | -4.10 |
|  | Hyoscyamine | ERBB2 | -3.67 |
|  | Hyoscyamine | ERBB4 | -3.68 |
|  | Hyoscyamine | FGFR1 | -3.25 |
|  | Hyoscyamine | GSK3B | -2.74 |
|  | Hyoscyamine | HSP90AA1 | -4.73 |
|  | Hyoscyamine | IGF1R | -3.21 |
|  | Hyoscyamine | IL2 | -3.38 |
|  | Hyoscyamine | INSR | -2.58 |
|  | Hyoscyamine | JAK2 | -3.84 |
|  | Hyoscyamine | KIT | -2.39 |
|  | Hyoscyamine | MAP2K1 | -4.41 |
|  | Hyoscyamine | MAPK1 | -4.35 |
|  | Hyoscyamine | MCL1 | -3.49 |
|  | Hyoscyamine | MTOR | -2.80 |
|  | Hyoscyamine | NOS3 | -3.13 |
|  | Hyoscyamine | NTRK1 | -2.69 |
|  | Hyoscyamine | PIK3CA | -3.24 |
|  | Hyoscyamine | PRKAA1 | -2.60 |
|  | Hyoscyamine | RXRA | -2.08 |
|  | Hyoscyamine | VEGFA | -3.33 |
|  | (+)-Hyoscyamine | AKT1 | -2.69 |
|  | (+)-Hyoscyamine | BCL2 | -3.99 |
|  | (+)-Hyoscyamine | CCND1 | -3.72 |
|  | (+)-Hyoscyamine | EGFR | -3.82 |
|  | (+)-Hyoscyamine | ERBB2 | -3.89 |
|  | (+)-Hyoscyamine | ERBB4 | -3.65 |
|  | (+)-Hyoscyamine | FGFR1 | -2.93 |
|  | (+)-Hyoscyamine | GSK3B | -2.85 |
|  | (+)-Hyoscyamine | HSP90AA1 | -4.61 |
|  | (+)-Hyoscyamine | IGF1R | -4.07 |
|  | (+)-Hyoscyamine | IL2 | -3.00 |
|  | (+)-Hyoscyamine | INSR | -3.33 |
|  | (+)-Hyoscyamine | JAK2 | -2.64 |
|  | (+)-Hyoscyamine | KIT | -3.80 |
|  | (+)-Hyoscyamine | MAP2K1 | -4.52 |
|  | (+)-Hyoscyamine | MAPK1 | -3.29 |
|  | (+)-Hyoscyamine | MCL1 | -2.49 |
|  | (+)-Hyoscyamine | MTOR | -3.22 |
|  | (+)-Hyoscyamine | NOS3 | -3.09 |
|  | (+)-Hyoscyamine | NTRK1 | -2.89 |
|  | (+)-Hyoscyamine | PIK3CA | -3.16 |
|  | (+)-Hyoscyamine | PRKAA1 | -3.08 |
|  | (+)-Hyoscyamine | RXRA | -2.26 |
|  | (+)-Hyoscyamine | VEGFA | -3.13 |
|  | Quercetin | AKT1 | -2.74 |
|  | Quercetin | BCL2 | -3.04 |
|  | Quercetin | CCND1 | -2.20 |
|  | Quercetin | EGFR | -3.87 |
|  | Quercetin | ERBB2 | -3.24 |
|  | Quercetin | ERBB4 | -2.84 |
|  | Quercetin | FGFR1 | -2.98 |
|  | Quercetin | GSK3B | -2.51 |
|  | Quercetin | HSP90AA1 | -4.10 |
|  | Quercetin | IGF1R | -3.83 |
|  | Quercetin | IL2 | -2.70 |
|  | Quercetin | INSR | -2.37 |
|  | Quercetin | JAK2 | -2.78 |
|  | Quercetin | KIT | -2.59 |
|  | Quercetin | MAP2K1 | -4.05 |
|  | Quercetin | MAPK1 | -2.54 |
|  | Quercetin | MCL1 | -2.51 |
|  | Quercetin | MTOR | -3.46 |
|  | Quercetin | NOS3 | -2.29 |
|  | Quercetin | NTRK1 | -1.85 |
|  | Quercetin | PIK3CA | -3.13 |
|  | Quercetin | PRKAA1 | -2.43 |
|  | Quercetin | RXRA | -2.25 |
|  | Quercetin | VEGFA | -2.15 |
|  | (Z,S)-Jasmololone | AKT1 | -3.21 |
|  | (Z,S)-Jasmololone | BCL2 | -3.34 |
|  | (Z,S)-Jasmololone | CCND1 | -3.31 |
|  | (Z,S)-Jasmololone | EGFR | -3.79 |
|  | (Z,S)-Jasmololone | ERBB2 | -3.70 |
|  | (Z,S)-Jasmololone | ERBB4 | -3.23 |
|  | (Z,S)-Jasmololone | FGFR1 | -3.52 |
|  | (Z,S)-Jasmololone | GSK3B | -3.20 |
|  | (Z,S)-Jasmololone | HSP90AA1 | -4.29 |
|  | (Z,S)-Jasmololone | IGF1R | -3.33 |
|  | (Z,S)-Jasmololone | IL2 | -3.02 |
|  | (Z,S)-Jasmololone | INSR | -2.92 |
|  | (Z,S)-Jasmololone | JAK2 | -3.53 |
|  | (Z,S)-Jasmololone | KIT | -2.98 |
|  | (Z,S)-Jasmololone | MAP2K1 | -4.49 |
|  | (Z,S)-Jasmololone | MAPK1 | -3.72 |
|  | (Z,S)-Jasmololone | MCL1 | -3.01 |
|  | (Z,S)-Jasmololone | MTOR | -3.00 |
|  | (Z,S)-Jasmololone | NOS3 | -2.84 |
|  | (Z,S)-Jasmololone | NTRK1 | -2.64 |
|  | (Z,S)-Jasmololone | PIK3CA | -2.70 |
|  | (Z,S)-Jasmololone | PRKAA1 | -2.78 |
|  | (Z,S)-Jasmololone | RXRA | -2.78 |
|  | (Z,S)-Jasmololone | VEGFA | -3.87 |
|  | Jasmolone | AKT1 | -2.96 |
|  | Jasmolone | BCL2 | -3.43 |
|  | Jasmolone | CCND1 | -3.39 |
|  | Jasmolone | EGFR | -4.06 |
|  | Jasmolone | ERBB2 | -3.79 |
|  | Jasmolone | ERBB4 | -2.95 |
|  | Jasmolone | FGFR1 | 3.92 |
|  | Jasmolone | GSK3B | -3.17 |
|  | Jasmolone | HSP90AA1 | -3.70 |
|  | Jasmolone | IGF1R | -3.02 |
|  | Jasmolone | IL2 | -2.97 |
|  | Jasmolone | INSR | -3.02 |
|  | Jasmolone | JAK2 | -2.84 |
|  | Jasmolone | KIT | -2.73 |
|  | Jasmolone | MAP2K1 | -4.71 |
|  | Jasmolone | MAPK1 | -3.31 |
|  | Jasmolone | MCL1 | -3.28 |
|  | Jasmolone | MTOR | -3.05 |
|  | Jasmolone | NOS3 | -3.04 |
|  | Jasmolone | NTRK1 | -3.00 |
|  | Jasmolone | PIK3CA | -2.67 |
|  | Jasmolone | PRKAA1 | -2.79 |
|  | Jasmolone | RXRA | -3.49 |
|  | Jasmolone | VEGFA | -3.23 |
|  | 2'-Hydroxymethylophiopogonone A | AKT1 | -3.22 |
|  | 2'-Hydroxymethylophiopogonone A | BCL2 | -2.68 |
|  | 2'-Hydroxymethylophiopogonone A | CCND1 | -2.81 |
|  | 2'-Hydroxymethylophiopogonone A | EGFR | -3.82 |
|  | 2'-Hydroxymethylophiopogonone A | ERBB2 | -3.46 |
|  | 2'-Hydroxymethylophiopogonone A | ERBB4 | -2.74 |
|  | 2'-Hydroxymethylophiopogonone A | FGFR1 | -3.70 |
|  | 2'-Hydroxymethylophiopogonone A | GSK3B | -3.14 |
|  | 2'-Hydroxymethylophiopogonone A | HSP90AA1 | -3.69 |
|  | 2'-Hydroxymethylophiopogonone A | IGF1R | -2.39 |
|  | 2'-Hydroxymethylophiopogonone A | IL2 | -2.77 |
|  | 2'-Hydroxymethylophiopogonone A | INSR | -2.56 |
|  | 2'-Hydroxymethylophiopogonone A | JAK2 | -2.60 |
|  | 2'-Hydroxymethylophiopogonone A | KIT | -2.53 |
|  | 2'-Hydroxymethylophiopogonone A | MAP2K1 | -4.86 |
|  | 2'-Hydroxymethylophiopogonone A | MAPK1 | -3.27 |
|  | 2'-Hydroxymethylophiopogonone A | MCL1 | -2.46 |
|  | 2'-Hydroxymethylophiopogonone A | MTOR | -2.30 |
|  | 2'-Hydroxymethylophiopogonone A | NOS3 | -2.80 |
|  | 2'-Hydroxymethylophiopogonone A | NTRK1 | -3.47 |
|  | 2'-Hydroxymethylophiopogonone A | PIK3CA | -2.93 |
|  | 2'-Hydroxymethylophiopogonone A | PRKAA1 | -2.20 |
|  | 2'-Hydroxymethylophiopogonone A | RXRA | -2.20 |
|  | 2'-Hydroxymethylophiopogonone A | VEGFA | -2.92 |
|  | Cnidilin | AKT1 | -3.71 |
|  | Cnidilin | BCL2 | -4.76 |
|  | Cnidilin | CCND1 | -3.38 |
|  | Cnidilin | EGFR | -4.54 |
|  | Cnidilin | ERBB2 | -4.24 |
|  | Cnidilin | ERBB4 | -3.33 |
|  | Cnidilin | FGFR1 | -3.48 |
|  | Cnidilin | GSK3B | -2.81 |
|  | Cnidilin | HSP90AA1 | -4.15 |
|  | Cnidilin | IGF1R | -3.66 |
|  | Cnidilin | IL2 | -2.94 |
|  | Cnidilin | INSR | -3.71 |
|  | Cnidilin | JAK2 | -2.81 |
|  | Cnidilin | KIT | -3.15 |
|  | Cnidilin | MAP2K1 | -4.61 |
|  | Cnidilin | MAPK1 | -4.02 |
|  | Cnidilin | MCL1 | -2.82 |
|  | Cnidilin | MTOR | -3.35 |
|  | Cnidilin | NOS3 | -3.61 |
|  | Cnidilin | NTRK1 | -3.19 |
|  | Cnidilin | PIK3CA | -3.57 |
|  | Cnidilin | PRKAA1 | -3.94 |
|  | Cnidilin | RXRA | -3.92 |
|  | Cnidilin | VEGFA | -4.19 |
|  | Ophiopogonanone B | AKT1 | -2.77 |
|  | Ophiopogonanone B | BCL2 | -3.47 |
|  | Ophiopogonanone B | CCND1 | -2.64 |
|  | Ophiopogonanone B | EGFR | -4.31 |
|  | Ophiopogonanone B | ERBB2 | -3.98 |
|  | Ophiopogonanone B | ERBB4 | -2.92 |
|  | Ophiopogonanone B | FGFR1 | -3.06 |
|  | Ophiopogonanone B | GSK3B | -2.90 |
|  | Ophiopogonanone B | HSP90AA1 | -3.72 |
|  | Ophiopogonanone B | IGF1R | -2.92 |
|  | Ophiopogonanone B | IL2 | -2.92 |
|  | Ophiopogonanone B | INSR | -2.48 |
|  | Ophiopogonanone B | JAK2 | -2.85 |
|  | Ophiopogonanone B | KIT | -2.44 |
|  | Ophiopogonanone B | MAP2K1 | -4.60 |
|  | Ophiopogonanone B | MAPK1 | -2.66 |
|  | Ophiopogonanone B | MCL1 | -2.98 |
|  | Ophiopogonanone B | MTOR | -3.40 |
|  | Ophiopogonanone B | NOS3 | -3.53 |
|  | Ophiopogonanone B | NTRK1 | -3.61 |
|  | Ophiopogonanone B | PIK3CA | -3.19 |
|  | Ophiopogonanone B | PRKAA1 | -3.11 |
|  | Ophiopogonanone B | RXRA | -2.87 |
|  | Ophiopogonanone B | VEGFA | -3.03 |
|  | (S)-p-Coumaroyloctopamine | AKT1 | -2.29 |
|  | (S)-p-Coumaroyloctopamine | BCL2 | -2.56 |
|  | (S)-p-Coumaroyloctopamine | CCND1 | -2.58 |
|  | (S)-p-Coumaroyloctopamine | EGFR | -4.45 |
|  | (S)-p-Coumaroyloctopamine | ERBB2 | -5.53 |
|  | (S)-p-Coumaroyloctopamine | ERBB4 | -2.97 |
|  | (S)-p-Coumaroyloctopamine | FGFR1 | -2.62 |
|  | (S)-p-Coumaroyloctopamine | GSK3B | -2.95 |
|  | (S)-p-Coumaroyloctopamine | HSP90AA1 | -4.03 |
|  | (S)-p-Coumaroyloctopamine | IGF1R | -2.37 |
|  | (S)-p-Coumaroyloctopamine | IL2 | -2.86 |
|  | (S)-p-Coumaroyloctopamine | INSR | -2.51 |
|  | (S)-p-Coumaroyloctopamine | JAK2 | -2.46 |
|  | (S)-p-Coumaroyloctopamine | KIT | -2.29 |
|  | (S)-p-Coumaroyloctopamine | MAP2K1 | -5.93 |
|  | (S)-p-Coumaroyloctopamine | MAPK1 | -3.09 |
|  | (S)-p-Coumaroyloctopamine | MCL1 | -2.74 |
|  | (S)-p-Coumaroyloctopamine | MTOR | -2.20 |
|  | (S)-p-Coumaroyloctopamine | NOS3 | -2.94 |
|  | (S)-p-Coumaroyloctopamine | NTRK1 | -2.36 |
|  | (S)-p-Coumaroyloctopamine | PIK3CA | -2.28 |
|  | (S)-p-Coumaroyloctopamine | PRKAA1 | -2.14 |
|  | (S)-p-Coumaroyloctopamine | RXRA | -1.86 |
|  | (S)-p-Coumaroyloctopamine | VEGFA | -2.77 |
|  | Melianone | AKT1 | -4.55 |
|  | Melianone | BCL2 | -4.85 |
|  | Melianone | CCND1 | -5.11 |
|  | Melianone | EGFR | -6.25 |
|  | Melianone | ERBB2 | -7.40 |
|  | Melianone | ERBB4 | -4.28 |
|  | Melianone | FGFR1 | -4.96 |
|  | Melianone | GSK3B | -4.81 |
|  | Melianone | HSP90AA1 | -6.75 |
|  | Melianone | IGF1R | -4.52 |
|  | Melianone | IL2 | -4.71 |
|  | Melianone | INSR | -4.89 |
|  | Melianone | JAK2 | -4.88 |
|  | Melianone | KIT | -4.53 |
|  | Melianone | MAP2K1 | -7.07 |
|  | Melianone | MAPK1 | -4.43 |
|  | Melianone | MCL1 | -4.74 |
|  | Melianone | MTOR | -5.19 |
|  | Melianone | NOS3 | -5.72 |
|  | Melianone | NTRK1 | -4.79 |
|  | Melianone | PIK3CA | -4.89 |
|  | Melianone | PRKAA1 | -4.61 |
|  | Melianone | RXRA | -4.73 |
|  | Melianone | VEGFA | -5.29 |
| YGJ-BBB | Orchinol | AKT1 | -3.74 |
|  | Orchinol | BCL2 | -3.21 |
|  | Orchinol | BRAF | -3.84 |
|  | Orchinol | CCND1 | -3.62 |
|  | Orchinol | EGFR | -4.08 |
|  | Orchinol | ERBB2 | -5.16 |
|  | Orchinol | ESR1 | -3.39 |
|  | Orchinol | ESR2 | -3.34 |
|  | Orchinol | IGF1R | -3.72 |
|  | Orchinol | MAP2K1 | -4.56 |
|  | Orchinol | MAPK1 | -3.25 |
|  | Orchinol | MMP9 | -3.77 |
|  | Orchinol | MTOR | -3.47 |
|  | Orchinol | PIK3CA | -3.71 |
|  | N-coumaroyltyramine | AKT1 | -2.92 |
|  | N-coumaroyltyramine | BCL2 | -3.80 |
|  | N-coumaroyltyramine | BRAF | -3.07 |
|  | N-coumaroyltyramine | CCND1 | -2.80 |
|  | N-coumaroyltyramine | EGFR | -5.25 |
|  | N-coumaroyltyramine | ERBB2 | -4.59 |
|  | N-coumaroyltyramine | ESR1 | -2.91 |
|  | N-coumaroyltyramine | ESR2 | -2.94 |
|  | N-coumaroyltyramine | IGF1R | -3.42 |
|  | N-coumaroyltyramine | MAP2K1 | -5.14 |
|  | N-coumaroyltyramine | MAPK1 | -5.09 |
|  | N-coumaroyltyramine | MMP9 | -2.76 |
|  | N-coumaroyltyramine | MTOR | -3.33 |
|  | N-coumaroyltyramine | PIK3CA | -2.92 |
|  | Hyoscyamine | AKT1 | -2.51 |
|  | Hyoscyamine | BCL2 | -3.10 |
|  | Hyoscyamine | BRAF | -4.74 |
|  | Hyoscyamine | CCND1 | -3.23 |
|  | Hyoscyamine | EGFR | -4.10 |
|  | Hyoscyamine | ERBB2 | -3.67 |
|  | Hyoscyamine | ESR1 | -3.13 |
|  | Hyoscyamine | ESR2 | -3.13 |
|  | Hyoscyamine | IGF1R | -3.21 |
|  | Hyoscyamine | MAP2K1 | -4.41 |
|  | Hyoscyamine | MAPK1 | -4.35 |
|  | Hyoscyamine | MMP9 | -3.21 |
|  | Hyoscyamine | MTOR | -2.80 |
|  | Hyoscyamine | PIK3CA | -3.24 |
|  | (+)-Hyoscyamine | AKT1 | -2.69 |
|  | (+)-Hyoscyamine | BCL2 | -3.99 |
|  | (+)-Hyoscyamine | BRAF | -2.80 |
|  | (+)-Hyoscyamine | CCND1 | -3.72 |
|  | (+)-Hyoscyamine | EGFR | -3.82 |
|  | (+)-Hyoscyamine | ERBB2 | -3.89 |
|  | (+)-Hyoscyamine | ESR1 | -3.42 |
|  | (+)-Hyoscyamine | ESR2 | -2.74 |
|  | (+)-Hyoscyamine | IGF1R | -4.07 |
|  | (+)-Hyoscyamine | MAP2K1 | -4.52 |
|  | (+)-Hyoscyamine | MAPK1 | -3.29 |
|  | (+)-Hyoscyamine | MMP9 | -3.06 |
|  | (+)-Hyoscyamine | MTOR | -3.22 |
|  | (+)-Hyoscyamine | PIK3CA | -3.16 |
|  | (Z,S)-Jasmololone | AKT1 | -3.21 |
|  | (Z,S)-Jasmololone | BCL2 | -3.34 |
|  | (Z,S)-Jasmololone | BRAF | -3.05 |
|  | (Z,S)-Jasmololone | CCND1 | -3.31 |
|  | (Z,S)-Jasmololone | EGFR | -3.79 |
|  | (Z,S)-Jasmololone | ERBB2 | -3.70 |
|  | (Z,S)-Jasmololone | ESR1 | -2.89 |
|  | (Z,S)-Jasmololone | ESR2 | -2.91 |
|  | (Z,S)-Jasmololone | IGF1R | -3.33 |
|  | (Z,S)-Jasmololone | MAP2K1 | -4.49 |
|  | (Z,S)-Jasmololone | MAPK1 | -3.72 |
|  | (Z,S)-Jasmololone | MMP9 | -3.88 |
|  | (Z,S)-Jasmololone | MTOR | -3.00 |
|  | (Z,S)-Jasmololone | PIK3CA | -2.70 |
|  | Jasmolone | AKT1 | -2.96 |
|  | Jasmolone | BCL2 | -3.43 |
|  | Jasmolone | BRAF | -3.93 |
|  | Jasmolone | CCND1 | -3.39 |
|  | Jasmolone | EGFR | -4.06 |
|  | Jasmolone | ERBB2 | -3.79 |
|  | Jasmolone | ESR1 | -2.77 |
|  | Jasmolone | ESR2 | -2.98 |
|  | Jasmolone | IGF1R | -3.02 |
|  | Jasmolone | MAP2K1 | -4.71 |
|  | Jasmolone | MAPK1 | -3.31 |
|  | Jasmolone | MMP9 | -3.53 |
|  | Jasmolone | MTOR | -3.05 |
|  | Jasmolone | PIK3CA | -2.67 |
|  | Cnidilin | AKT1 | -3.71 |
|  | Cnidilin | BCL2 | -4.76 |
|  | Cnidilin | BRAF | -3.25 |
|  | Cnidilin | CCND1 | -3.38 |
|  | Cnidilin | EGFR | -4.54 |
|  | Cnidilin | ERBB2 | -4.24 |
|  | Cnidilin | ESR1 | -3.85 |
|  | Cnidilin | ESR2 | -3.52 |
|  | Cnidilin | IGF1R | -3.66 |
|  | Cnidilin | MAP2K1 | -4.61 |
|  | Cnidilin | MAPK1 | -4.02 |
|  | Cnidilin | MMP9 | -4.61 |
|  | Cnidilin | MTOR | -3.35 |
|  | Cnidilin | PIK3CA | -3.57 |
|  | Ophiopogonanone B | AKT1 | -3.09 |
|  | Ophiopogonanone B | BCL2 | -2.33 |
|  | Ophiopogonanone B | BRAF | -3.30 |
|  | Ophiopogonanone B | CCND1 | -2.46 |
|  | Ophiopogonanone B | EGFR | -4.23 |
|  | Ophiopogonanone B | ERBB2 | -4.27 |
|  | Ophiopogonanone B | ESR1 | -2.83 |
|  | Ophiopogonanone B | ESR2 | -2.71 |
|  | Ophiopogonanone B | IGF1R | -2.96 |
|  | Ophiopogonanone B | MAP2K1 | -3.76 |
|  | Ophiopogonanone B | MAPK1 | -2.73 |
|  | Ophiopogonanone B | MMP9 | -3.83 |
|  | Ophiopogonanone B | MTOR | -2.87 |
|  | Ophiopogonanone B | PIK3CA | -3.00 |
|  | Bergaptin | AKT1 | -3.60 |
|  | Bergaptin | BCL2 | -3.46 |
|  | Bergaptin | BRAF | -3.66 |
|  | Bergaptin | CCND1 | -3.34 |
|  | Bergaptin | EGFR | -4.41 |
|  | Bergaptin | ERBB2 | -5.78 |
|  | Bergaptin | ESR1 | -3.32 |
|  | Bergaptin | ESR2 | -3.71 |
|  | Bergaptin | IGF1R | -4.28 |
|  | Bergaptin | MAP2K1 | -5.55 |
|  | Bergaptin | MAPK1 | -5.33 |
|  | Bergaptin | MMP9 | -4.08 |
|  | Bergaptin | MTOR | -3.31 |
|  | Bergaptin | PIK3CA | -4.02 |
|  | Ammidin | AKT1 | -3.84 |
|  | Ammidin | BCL2 | -3.97 |
|  | Ammidin | BRAF | -5.01 |
|  | Ammidin | CCND1 | -3.48 |
|  | Ammidin | EGFR | -4.67 |
|  | Ammidin | ERBB2 | -5.93 |
|  | Ammidin | ESR1 | -4.20 |
|  | Ammidin | ESR2 | -4.57 |
|  | Ammidin | IGF1R | -4.11 |
|  | Ammidin | MAP2K1 | -5.06 |
|  | Ammidin | MAPK1 | -4.51 |
|  | Ammidin | MMP9 | -4.47 |
|  | Ammidin | MTOR | -4.54 |
|  | Ammidin | PIK3CA | -4.58 |
|  | Ruscogenin | AKT1 | -4.97 |
|  | Ruscogenin | BCL2 | -5.56 |
|  | Ruscogenin | BRAF | -5.28 |
|  | Ruscogenin | CCND1 | -4.77 |
|  | Ruscogenin | EGFR | -8.37 |
|  | Ruscogenin | ERBB2 | -5.66 |
|  | Ruscogenin | ESR1 | -5.49 |
|  | Ruscogenin | ESR2 | -5.28 |
|  | Ruscogenin | IGF1R | -5.32 |
|  | Ruscogenin | MAP2K1 | -6.96 |
|  | Ruscogenin | MAPK1 | -4.75 |
|  | Ruscogenin | MMP9 | -4.69 |
|  | Ruscogenin | MTOR | -6.67 |
|  | Ruscogenin | PIK3CA | -4.66 |
|  | Methylophiopogonanone B | AKT1 | -2.87 |
|  | Methylophiopogonanone B | BCL2 | -2.51 |
|  | Methylophiopogonanone B | BRAF | -3.31 |
|  | Methylophiopogonanone B | CCND1 | -2.71 |
|  | Methylophiopogonanone B | EGFR | -4.36 |
|  | Methylophiopogonanone B | ERBB2 | -4.61 |
|  | Methylophiopogonanone B | ESR1 | -2.84 |
|  | Methylophiopogonanone B | ESR2 | -2.95 |
|  | Methylophiopogonanone B | IGF1R | -3.36 |
|  | Methylophiopogonanone B | MAP2K1 | -4.26 |
|  | Methylophiopogonanone B | MAPK1 | -3.09 |
|  | Methylophiopogonanone B | MMP9 | -2.94 |
|  | Methylophiopogonanone B | MTOR | -4.16 |
|  | Methylophiopogonanone B | PIK3CA | -2.91 |
